# Reactive Oxygen Species Drive Cell Migration and PD-L1 Expression via YB-1 Phosphorylation in Pleural Mesothelioma

**DOI:** 10.3390/antiox15010121

**Published:** 2026-01-17

**Authors:** Muhammad Hashim, Gerald Timelthaler, Dominik Kirchhofer, Beatrice Irina Kudlacek, Berta Mosleh, Katharina Sinn, Ezzat Mohamed Awad, Mir Alireza Hoda, Bettina Grasl-Kraupp, Balazs Dome, Walter Berger, Georg Krupitza, Karin Schelch, Michael Grusch

**Affiliations:** 1 Center for Cancer Research and Comprehensive Cancer Center Vienna, Medical University of Vienna, 1090 Vienna, Austria; mhashim@bs.qau.edu.pk (M.H.);; 2 Department of Thoracic Surgery, Comprehensive Cancer Center Vienna, Medical University of Vienna, 1090 Vienna, Austriamir.hoda@meduniwien.ac.at (M.A.H.);; 3 Institute of Specific Prophylaxis and Tropical Medicine, Center for Pathophysiology, Infectiology and Immunology, Ocular Immunology & Infectiology, Medical University of Vienna, 1090 Vienna, Austria; ezzat.awad@meduniwien.ac.at; 4 National Koranyi Institute of Pulmonology, 1121 Budapest, Hungary; 5 Department of Thoracic Surgery, Semmelweis University and National Institute of Oncology, 1122 Budapest, Hungary; 6 Department of Translational Medicine, Lund University, 221 00 Lund, Sweden; 7 Department of Pathology, Medical University of Vienna, 1090 Vienna, Austria

**Keywords:** pleural mesothelioma, ROS, cell migration, cell signalling, immune checkpoint proteins

## Abstract

Reactive oxygen species (ROS)-induced aberrant oncogenic signalling has been proposed to mediate the progression and development of pleural mesothelioma (PM). In this study, we demonstrate how ROS promote oncogenic signalling, especially in the context of cell migration and immune evasion via YB-1 phosphorylation in mesothelial and PM cell models. Xanthine (X)- and xanthine oxidase (XO)-generated ROS exposure led to increased migration and a more elongated cell shape in mesothelial and PM cells in live-cell videomicroscopy analyses. These effects were associated with the enhanced phosphorylation of ERK, AKT, and YB-1 and the elevated gene expression of PD-L1 and PD-L2, which were analysed with immunoblotting and quantitative real-time RT-PCR, respectively. The pharmacological inhibition of AKT (ipatasertib), MEK (trametinib), and RSK (BI-D1870) resulted in the reversal of ROS-induced effects, with the strongest effects observed upon the inhibition of YB-1 phosphorylation by BI-D1870. The results suggest that ROS exposure has a strong impact on cell migration and immune evasion not only in PM cells but also in mesothelial cells, from which PM arises. Interfering with ROS-responsive kinase pathways, particularly YB-1 phosphorylation, could counteract pro-migratory and immune-evasive effects in PM.

## 1. Introduction

Pleural mesothelioma (PM) is a devastating malignancy arising from mesothelial cells lining the pleural surfaces of the lungs and chest wall [1]. The median survival time of patients is little more than one year and has not seen major improvements in the last decade, despite the addition of immunotherapy and, more recently, chemo-immunotherapy to the therapeutic arsenal [2,3]. In the majority of cases, PM is caused by the inhalation of asbestos fibres, which penetrate the lung epithelium and are deposited in the pleural space where they can persist for a prolonged period, leading to chronic inflammation and tissue damage [1]. An essential factor in PM pathogenesis is attributed to reactive oxygen species (ROS) that are generated at iron-containing fibre surfaces via the Fenton reaction [4,5]. Another source of ROS during PM development is the failed attempts of macrophages to digest the asbestos fibres, a process that has been described as frustrated phagocytosis [4,5]. While many countries have enacted bans or severe restrictions on the use of asbestos, its mining and use continue in several others. Even in countries with strict asbestos regulations, exposure can still occur during demolition and renovation work of older buildings or as a consequence of disasters such as the collapse of the World Trade Center in New York City in 2001. Moreover, similar ROS-generating and carcinogenic mechanisms to those of asbestos have been experimentally demonstrated for carbon nanotubes, which are increasingly being used in a wide range of applications, including batteries and pharmaceutical products [6]. ROS are important contributors to DNA damage, which leads to mutations in exposed cells [7]. However, the overall mutational burden of PM is relatively low, and it primarily involves mutations and deletions in tumour suppressor genes such as BAP1 (BRCA1-associated protein 1), methylthioadenosine phosphorylase (MTAP), CDKN2A (cyclin-dependent kinase inhibitor 2A), and TP53 [8]. ROS can also directly affect the activation of cellular signal transduction pathways, and the resulting alterations in cell behaviour may influence PM development and progression [5]. The MAPK/ERK (mitogen-activated protein kinase/extracellular signal-regulated kinase) and the AKT (protein kinase B) pathway are two key growth signalling pathways with demonstrated relevance in PM [9,10,11]. In addition, YB-1 (Y-box binding protein 1) is overexpressed in PM and contributes to the survival and migration of PM cells [12]. YB-1 is a nucleic acid-binding protein that regulates transcription, translation, and RNA splicing [13]. As a transcription factor, YB-1 enhanced the expression of EGFR (epidermal growth factor receptor) in breast cancer [14]. Importantly, YB-1 was also shown to increase the expression of PD-L1 (programmed death ligand 1) in liver and lung cancer by acting as a transcription regulator at the PD-L1 promoter [15,16,17]. PD-L1 is a critical immune checkpoint protein (ICP), and its expression in cancer cells contributes to immune evasion [18]. Moreover, PD-L1 is a key target for immunotherapies that are used for the treatment of an increasing number of malignancies including PM [3,19,20]. The upregulation of PD-L1 by ROS has been reported in lung cancer and pancreatic cancer and involved c-Myc and FGFR1 (fibroblast growth factor 1), respectively [21,22], but it has not been investigated in PM.

The goal of this study was to investigate the effects of ROS on the migration, signalling, and ICP expression of mesothelial cells and PM cells. For this purpose, we exposed cultured cells to xanthine (X) and xanthine oxidase (XO). XO catalyses the oxidation of xanthine to uric acid, thereby generating superoxide anions and hydrogen peroxide, which has previously been used as a model for subjecting cells to oxidative stress [23,24,25]. This model was used for our investigation, since the presence of both superoxide anions and hydrogen peroxide reflects the exposure of mesothelial and PM cells during PM development in vivo [4,5]. We analysed the impact of the generated ROS on cell migration and cell morphology, stimulation of MAPK and AKT signalling, protein expression and phosphorylation of YB-1, and gene expression of ICPs. Pharmacological inhibitors of the respective signalling pathways were used to explore their relevance for ROS-induced effects.

## 2. Material and Methods

### 2.1. Cell Culture

The human PM cell line MSTO-211H and the non-malignant human mesothelial cell line Met5A were obtained from the American Type Culture Collection (ATCC, Rockville, MD, USA). The SPC212 PM cell line was provided by Prof. R. Stahel (University of Zürich, Zürich, Switzerland). NP2i is an immortalised form of the NP2 primary mesothelial cells. The establishment of NP2, immortalization with hTERT, and comparison of the immortalised cells with their primary cell counterparts have been previously described [26]. Cells were cultured in RPMI-1640 medium supplemented with 10% foetal bovine serum (FBS) in a humidified incubator at 37 °C and 5% CO_2_. Cells were authenticated as described and regularly checked for *Mycoplasma* infection [26,27,28]. Unless stated otherwise, 5 × 10^5^ cells per well were seeded for experiments into 6-well plates 24 h before the start of the experiment to achieve a confluence of 70–80%.

### 2.2. Chemicals and Reagents

Xanthine (X) and xanthine oxidase (XO) were purchased from Sigma-Aldrich (St. Louis, MO, USA). The ROS-detecting reagent DCFDA (2′,7′-dichlorodihydrofluorescein diacetate, Cellular ROS Assay Kit, Cat. No. ab113851) was obtained from Abcam (Cambridge, UK). Primary antibodies against ERK1/2 (Cat. No. 4695S), phospho-ERK1/2 (Cat. No. 9101), AKT (Cat. No. 4691), phospho-AKT (Cat. No. 4060), phospho-YB-1 (Cat. No. 2900, C34A2), and GAPDH (Cat. No. 5174) were purchased from Cell Signaling Technology (Danvers, MA, USA). Anti-YB-1 antibody (Cat. No. ab12148) was purchased from Abcam (Cambridge, UK). HRP-conjugated rabbit secondary antibody (Cat. No. P0448) was obtained from Dako (Agilent Technologies, Santa Clara, CA, USA). The RSK inhibitor BI-D1870 (Cat. No. sc-397022A) was procured from Santa Cruz Biotechnology (Dallas, TX, USA). The MEK inhibitor trametinib (Cat. No. HY-10999), the AKT inhibitor ipatasertib (Cat. No. HY-15186), and the YB-1 inhibitor SU056 (Cat. No. HY-150231) were purchased from MedChemExpress (Monmouth Junction, NJ, USA). The Clarity Western ECL Substrate was procured from Bio-Rad (Cat. No. 170-5060, Hercules, CA, USA).

### 2.3. ROS Generation

Xanthine (X) was dissolved in 1 M NaOH at a concentration of 10 mg/mL. Xanthine oxidase (XO, 7 U/mg) was dissolved in 50 mM Tris-HCl at a concentration of 50 U/mL, corresponding to 10 mg/mL. To generate ROS, different concentrations of xanthine and xanthine oxidase (X/XO) were mixed in growth medium and immediately added to the cells at the indicated concentrations [23,24,25]. Further dilutions of X/XO and the vehicle were prepared in the growth medium. The vehicle contained only the solvents of X and XO, i.e., NaOH and Tris-HCl, respectively, which were used in final concentrations of 2 mM and 10 µM, respectively. The vehicle was used as a negative control in all experiments. Biological effects and signals were evaluated after 24 h to reduce potential indirect effects caused by ROS-induced mutations.

### 2.4. ROS Measurement

X/XO-generated ROS exposure in mesothelial and mesothelioma cells was measured using the DCFDA/H2DCFDA—Cellular ROS Assay Kit following the manufacturer’s protocol (Abcam, Cambridge, UK; Cat. No. ab113851). NP2i, Met5A, MSTO-211H, and SPC212 cells were seeded into 96-well plates (Greiner Bio-One, Kremsmünster, Austria, Cat. No. REF 655090) in growth medium without phenol red at a density of 2.5 × 10^4^ cells per well and allowed to adhere overnight. The DCFDA solution (20 µM) was prepared by diluting the 20 mM DCFDA stock solution in 10% FBS-supplemented dilution buffer. The growth medium was removed, and the cells were washed with the dilution buffer. Subsequently, 100 µL of 20 µM DCFDA solution was added to each well. Plates were covered with aluminium foil and incubated at 37 °C for 45 min to allow the DCFDA to enter the cells and be converted to DCFH (2′,7′-dichlorodihydrofluorescein) by cellular esterases. After the incubation period, the DCFDA solution was removed, the cells were washed with 100 µL dilution buffer, and fresh medium without phenol red was added to each well. Then, the cells were treated with the vehicle, different concentrations of X/XO, or tert-butyl hydroperoxide (TBHP), which was used as a positive control at a final concentration of 250 µM. The treated cells were incubated for 4 h. ROS induced oxidation of non-fluorscent DCFH to fluorescent DCF (2′,7′-dichlorofluorescein). Fluorescence intensity was measured immediately using a microplate reader (Tecan Austria GmbH, Groedig, Austria) at excitation/emission wavelengths of 485/535 nm. ROS levels were expressed as fluorescence intensity.

### 2.5. Cytotoxicity Detection

NP2i, Met5A, MSTO-211H, and SPC212 cells were seeded in 24-well plates at a density of 10^5^ cells per well to reach 70% confluence. To detect a potential cytotoxic effect of ROS exposure, cells were treated with X/XO at different concentrations for 24 h. On the next day, the cell culture supernatant was incubated with a dual-staining solution containing propidium iodide (PI) and Hoechst 33342 at final concentrations of 2 µg/mL and 5 µg/mL, respectively, for 1 h at 37 °C. Hoechst 33342 stained the nuclei of all cells for the assessment of nuclear morphology changes, whereas PI selectively labelled membrane-compromised (non-viable) cells. TBHP (50 µM) was used as positive control. Fluorescent images were captured using a Nikon Eclipse Ti inverted fluorescence microscope and a Nikon DS-Fi1c camera (Nikon Instrument Inc., Tokyo, Japan).

### 2.6. Inhibition of Signal Transduction Pathways

The pharmacological inhibitors of AKT (ipatasertib), MEK (trametinib), RSK (p90 ribosomal S6 kinase) (BI-D1870), and YB-1 (SU056) were dissolved in DMSO as 10 mM stocks. Cells were treated with either solvent (DMSO) or ipatasertib, trametinib, or BI-D1870 at a final concentration of 10 μM each together with X/XO (20/2 µg/mL) for 24 h or with SU056 at a final concentration of 3 µM for 72 h. Subsequent analyses were performed as described below.

### 2.7. Videomicroscopy and Analysis of Cell Migration

Cells were seeded in 6-well plates and on the next day treated with the vehicle or X/XO with or without pathway inhibitors at the indicated concentrations for 24 h. Videos were generated using an IncuCyte S3 live-cell analysis system (Sartorius AG, Göttingen, Germany). Images were taken every 30 min for 24 h. Cell migration was determined by manually tracking at least 50 individual cells using Fiji/ImageJ (v1.54p, NIH, Bethesda, MD, USA) to obtain x–y coordinates for each cell at all specific time points as described previously [27]. For the slower growing mesothelial cells, exclusion of dividing cells was done by manual inspection. For the rapidly dividing cancer cells, excluding dividing cells was not feasible, due to low numbers of cells not dividing within 24 h. For these cell models, in case of cell division, one daughter cell was arbitrarily selected for further tracking. In all cell models, 50 cells were randomly selected from three independent experiments and cells that died, exhibited ambiguous morphology, or migrated out of the microscopy window before the end of the observation period were excluded from analysis. These criteria were consistently applied across all experiments to minimize the potential observer bias of manual tracking analysis. For the further analysis of migratory behaviour, such as the visualisation of individual cell trajectories, origin plots were generated with the DiPer approach for cell migration analysis in Microsoft Excel [29]. For NP2i and MSTO-211H, DiPer approach was also used to analyse direction autocorrelation and average speed.

### 2.8. Cell Shape Analysis

For cell shape analysis, images were acquired using the IncuCyte S3 (Sartorius AG, Göttingen, Germany) live-cell imaging microscopy system over a 24 h treatment period. The treatment was performed using either the vehicle or X/XO with or without pathway inhibitors at the indicated concentrations. Images were analysed using Fiji/ImageJ by measuring cell shape parameters, including area, aspect ratio, and circularity. Individual cell outlines were drawn using the freehand selection tool, and the data were transferred to ROI manager. At least 30 individual cells per condition were analysed, and aspect ratios, circularity values, or circularity versus area plots are shown.

### 2.9. Protein Isolation and Immunoblotting

Cells were seeded in 6-well plates and left to adhere overnight. Then, media were replaced with 1 mL fresh RPMI-1640 supplemented with 10% FBS and treated as indicated for 24 h. Cells were then harvested in lysis buffer II (150 mM NaCl, 50 mM HEPES, 10% glycerol, 1 mM EDTA, 0.5 mM Na_3_VO_4_, 10 mM NaF, 1% Triton X-100, and 1.5 mM MgCl_2_) containing a protease inhibitor cocktail (Roche, Basel, Switzerland, Cat. No. 11697498001). Proteins were quantified using the BCA protein quantification assay (Bio-Rad, Cat. No. 5000006). Immunoblotting was performed as recently described [30]. After quantification, 10 µg of protein per lane was separated by SDS-PAGE and transferred onto PVDF membranes (Cytiva, Marlborough, MA, USA, Cat. No. 1060023) at 4 °C and 18 V overnight. The membranes were washed with 1× TBST and blocked for 1 h at room temperature in 3% BSA solution in TBST. PVDF membranes were then incubated overnight at 4 °C with the respective primary antibodies (ERK 1:1000, pERK 1:1000, AKT 1:000, pAKT 1:1000, YB-1 1:1000, pYB-1 1:500, and GAPDH 1:4000). On the next day, the membranes were washed with 1× TBST and incubated at room temperature with HRP-coupled secondary antibodies (1:10,000) in a BSA/TBST solution. Signals were developed using the Biorad Clarity Western ECL Substrate, and luminescent signals were recorded on CL-XPosure film (Thermo Scientific, Cat. No. 34089, Waltham, MA, USA). The bands were quantified using the Fiji/ImageJ gel analysis function.

### 2.10. RNA Isolation and qRT-PCR

Total RNA was isolated from the vehicle- and X/XO-treated cells using the InnuPREP Micro RNA kit (InnuScreen GmbH, Berlin, Germany) and reverse-transcribed with MLV-reverse transcriptase (Thermo Fisher Scientific, Waltham, MA, USA) according to the manufacturer’s protocol. Quantitative real-time reverse transcription (qRT)-PCR was performed for immune checkpoint genes (PD-L1/2, PVR, TNFRSF9, and VISTA) on a CFX96 Thermocycler (Bio-Rad, Hercules, CA, USA) using iTaq universal SYBR Green Supermix (Cat. # 1725124, Bio-Rad, USA) as described previously [27]. Primers for the immune checkpoint genes were designed with Clone Manager 9 (Scientific & Educational Software, Cary, NC, USA) or adopted from OriGene Technologies (Rockville, MD, USA). Primer sequences are listed in Supplementary Table S1. Differential gene expression levels normalised to the housekeeping gene GAPDH were calculated as 2^−^^ΔΔCt^ compared to the respective control.

### 2.11. Statistical Analysis

All experiments were performed independently in triplicate, and data were statistically analysed and plotted using GraphPad Prism 8.0.1 (GraphPad Software, Boston, MA, USA). Data are presented as mean ± standard deviation (SD), unless stated otherwise. Normality was assessed using Shapiro–Wilk test while homogeneity of variances was assessed using Brown-Forsythe test. Comparisons between treatment conditions were performed using one-way ANOVA followed by Dunnett’s test for multiple comparisons and unpaired *t*-test for comparison of two groups. A *p*-value < 0.05 was considered statistically significant.

## 3. Results

### 3.1. Treatment with Xanthine and Xanthine Oxidase Induces ROS Exposure of Mesothelial and PM Cells

**Figure 1:**
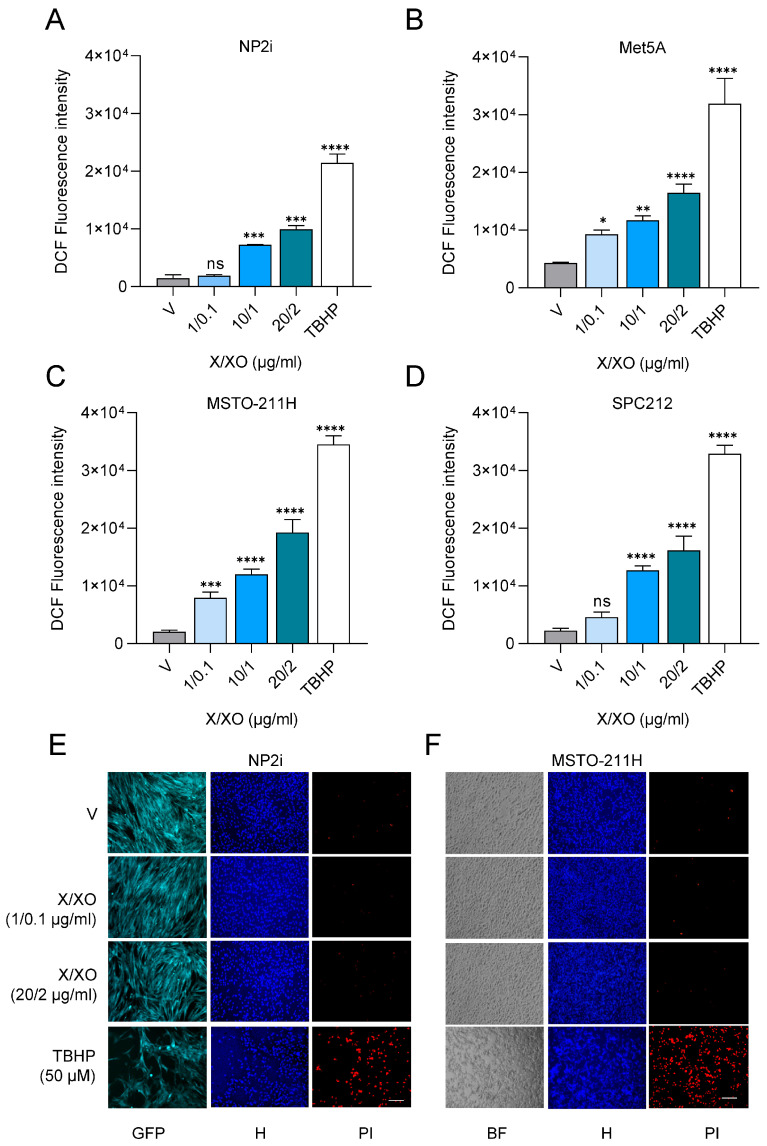
Treatment with xanthine and xanthine oxidase leads to generation of reactive oxygen species in mesothelial and mesothelioma cells. Bar graphs depicting DCF fluorescence intensity (arbitrary units) for mesothelial cells (**A**,**B**) and for pleural mesothelioma (**C**,**D**) cell lines after treatment with xanthine/xanthine oxidase (X/XO) at indicated concentrations compared to vehicle (V) and positive control (TBHP, 250 µM). Data from *n* = 3 experiments were analysed by one-way ANOVA followed by Dunnett’s test. Significance is indicated as ns *p* > 0.05, * *p* < 0.05, ** *p* < 0.01, *** *p* < 0.001, and **** *p* < 0.0001, treated versus vehicle. Error bars represent ± SD. Double staining with Hoechst 33342 and propidium iodide (PI) of mesothelial cells (**E**) and pleural mesothelioma cells (**F**) treated with vehicle (V) or 1/0.1 µg/mL and 20/2 µg/mL X/XO or 50 µM TBHP (as positive control) for 24 h. Hoechst 33342 (blue) shows nuclear morphology, and propidium iodide (PI, red) labels dead cells that have lost membrane integrity. Green fluorescence protein (GFP) and brightfield (BF) images are shown for NP2i and MSTO-211H, respectively. All microscopic images were taken at same magnification. Scale bar = 50 µm.

To subject two mesothelial (NP2i and Met5A) and two PM (MSTO-211H and SPC212) cell lines to ROS, we simultaneously co-treated them with increasing concentrations of xanthine (X) and xanthine oxidase (XO). The X/XO treatment resulted in a dose-dependent increase in intracellular ROS levels detected by 2′,7′-dichlorofluorescein (DCF) fluorescence after 4 h in both types of cell models (Figure 1A–D). The lowest concentration of 1 µg/mL X and 0.1 µg/mL XO and the highest concentration of 20 µg/mL X and 2 µg/mL XO were selected for further analysis. None of these concentrations resulted in cytotoxicity when analysed by double staining with Hoechst 33342 and propidium iodide after 24 h (Figure 1E,F, Supplementary Figure S1A,B).

### 3.2. ROS Increases Cell Migration and Alters Cell Shape

**Figure 2:**
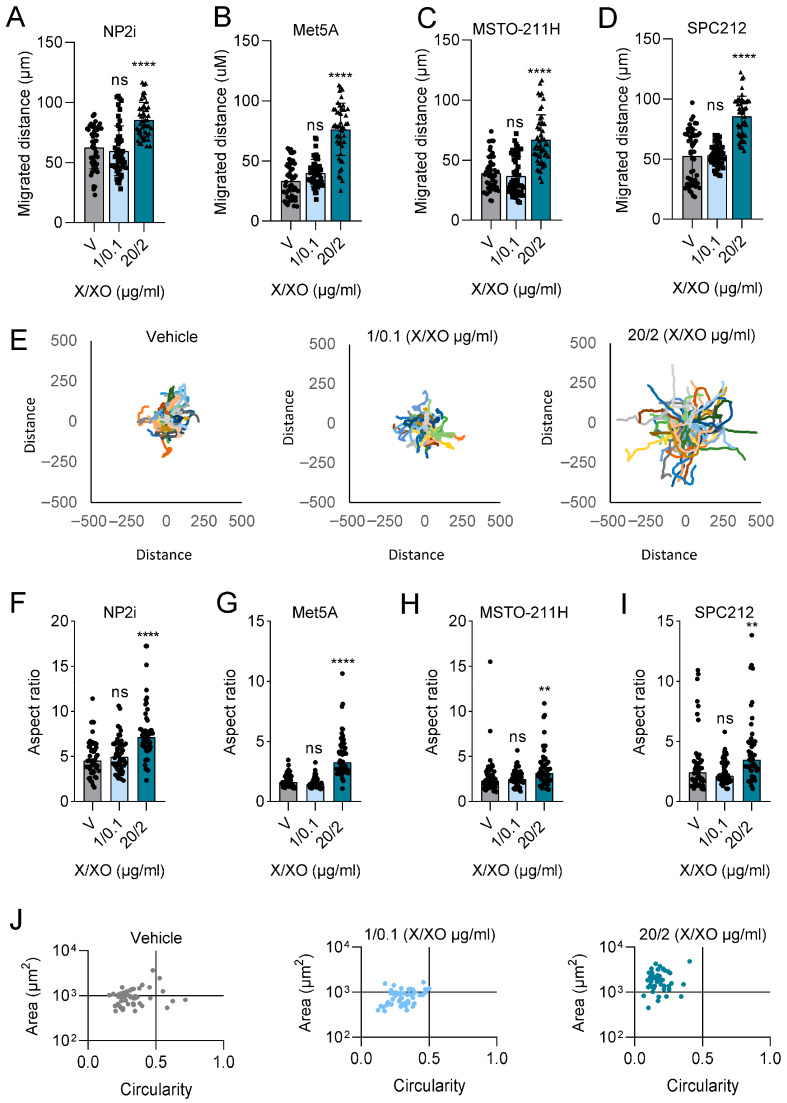
ROS induction increases cell migration and alters cell shape. Videomicroscopy was performed for mesothelial (**A**,**B**) and pleural mesothelioma (**C**,**D**) cells under treatment with xanthine/xanthine oxidase (X/XO) or vehicle (V) at indicated concentrations for 24 h. Migrated distances were assessed by manual cell tracking using Fiji/ImageJ. Bars represent mean migrated distance of at least 50 cells, and each dot represents one individual cell. Single origin plots of representative individual cell tracks are shown for NP2i (**E**). Aspect ratios (longer diameter/shorter diameter) of mesothelial (**F**,**G**) and pleural mesothelioma (**H**,**I**) were calculated from images taken after 24 h of treatment with X/XO or vehicle. Area and circularity under same treatment as above are shown for NP2i (**J**). Data from *n* = 3 experiments were analysed by one-way ANOVA followed by Dunnett’s test. Significance is indicated as ns *p* > 0.05, ** *p* < 0.01, and **** *p* < 0.0001, treated versus vehicle. Error bars represent ± SD.

During the experiments, we observed a change in cell morphology in response to ROS induction, and consequently analysed the impact of ROS on cell shape and cell migration. The results showed a significant increase in average migrated distances in all cell models 24 h after treatment with the higher concentration of 20 µg/mL X and 2 µg/mL XO (Figure 2A–E, Supplementary Figure S2A–C). In NP2i and MSTO-211H, directional persistence and average speed of migrating cells were determined in addition, and showed increased values after treatment with the higher X/XO concentration (Supplementary Figure S3A–D). Increased cell motility was accompanied by notable changes in cell morphology. Cells generally became more elongated, reflected in higher aspect ratios (longer diameter/shorter diameter) (Figure 2F–I), and became less circular and more spread out (indicated by a larger area) (Figure 2J, Supplementary Figure S4A–C).

### 3.3. ROS Treatment Leads to Phosphorylation of ERK, AKT, and YB-1

**Figure 3:**
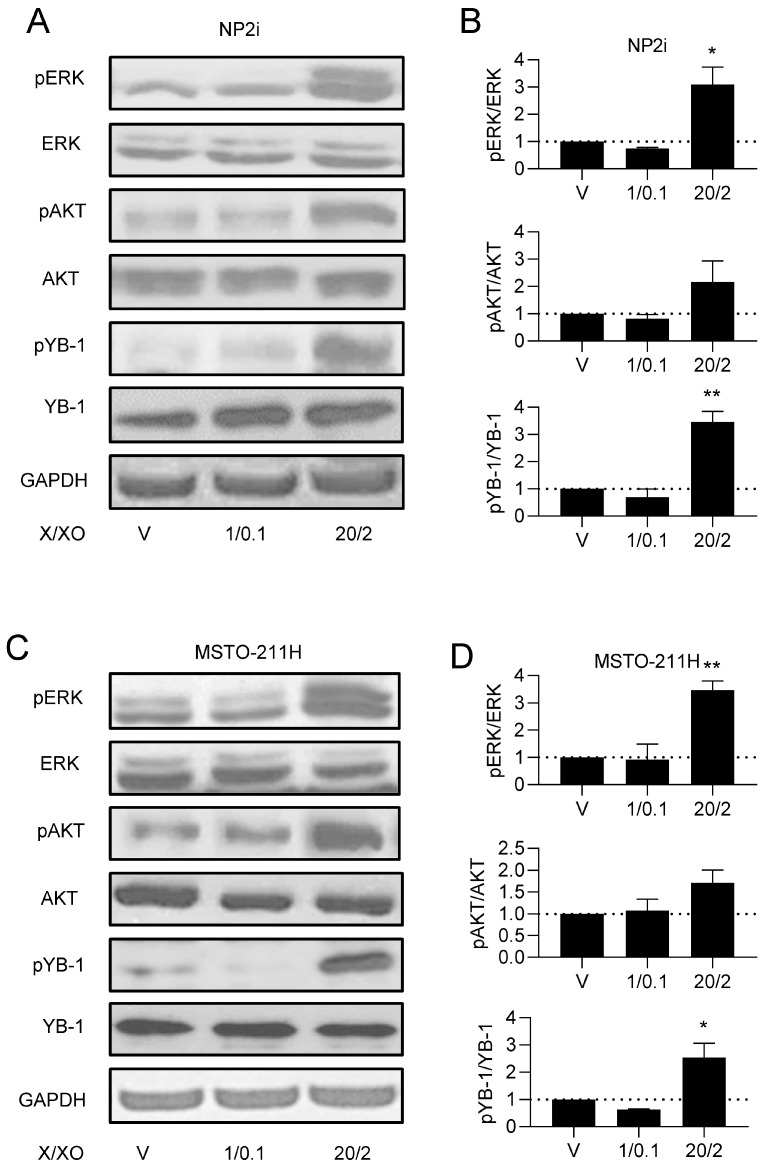
ROS treatment leads to phosphorylation of ERK, AKT, and YB-1. Representative examples (**A**,**C**) and quantification (**B**,**D**) of Western blots of phosphorylated (p) and total ERK, AKT, and YB-1 after treatment for 24 h with the indicated concentrations of xanthine/xanthine oxidase (X/XO, µg/mL) or vehicle (V). The housekeeping gene GAPDH was used as control for equal sample loading. Quantification data of *n* = 3 replicates are shown as phosphorylated-to-total protein ratios normalised to vehicle-treated samples set as 1. Data were analysed by one-way ANOVA followed by Dunnett’s test. Significance is indicated as * *p* < 0.05, ** *p* < 0.01, X/XO versus vehicle.

To identify signalling pathways that could mediate the ROS-induced cellular response, we analysed the MAPK and the phosphatidyl inositol 3-kinase (PI3K)/AKT pathways, two key cellular signalling pathways previously implicated in tumorigenesis and cell migration [31,32]. In agreement with the cell migration data, treatment with the higher concentration of X/XO resulted in the increased phosphorylation of ERK and AKT after 24 h of treatment. Expression levels of total ERK and total AKT remained unchanged compared to the housekeeping gene GAPDH. This effect was observed in both mesothelial cell lines (Figure 3A,B, Supplementary Figure S5A,B), as well as in the PM cells (Figure 3C,D, Supplementary Figure S5C,D). Since we had previously established YB-1 and its phosphorylation at serine 102 (pYB-1) as relevant factors in PM cell migration [27,33], we also analysed the expression of YB-1 and pYB-1. While YB-1 expression showed no change in response to the treatment, YB-1 serine 102 phosphorylation was increased by X/XO treatment at the higher concentration but not at the lower concentration in all investigated models (Figure 3A–D, Supplementary Figure S5A–D).

### 3.4. Inhibition of Signalling Pathways Can Reverse ROS-Induced Stimulation of Cell Migration and Cell Shape Changes

**Figure 4:**
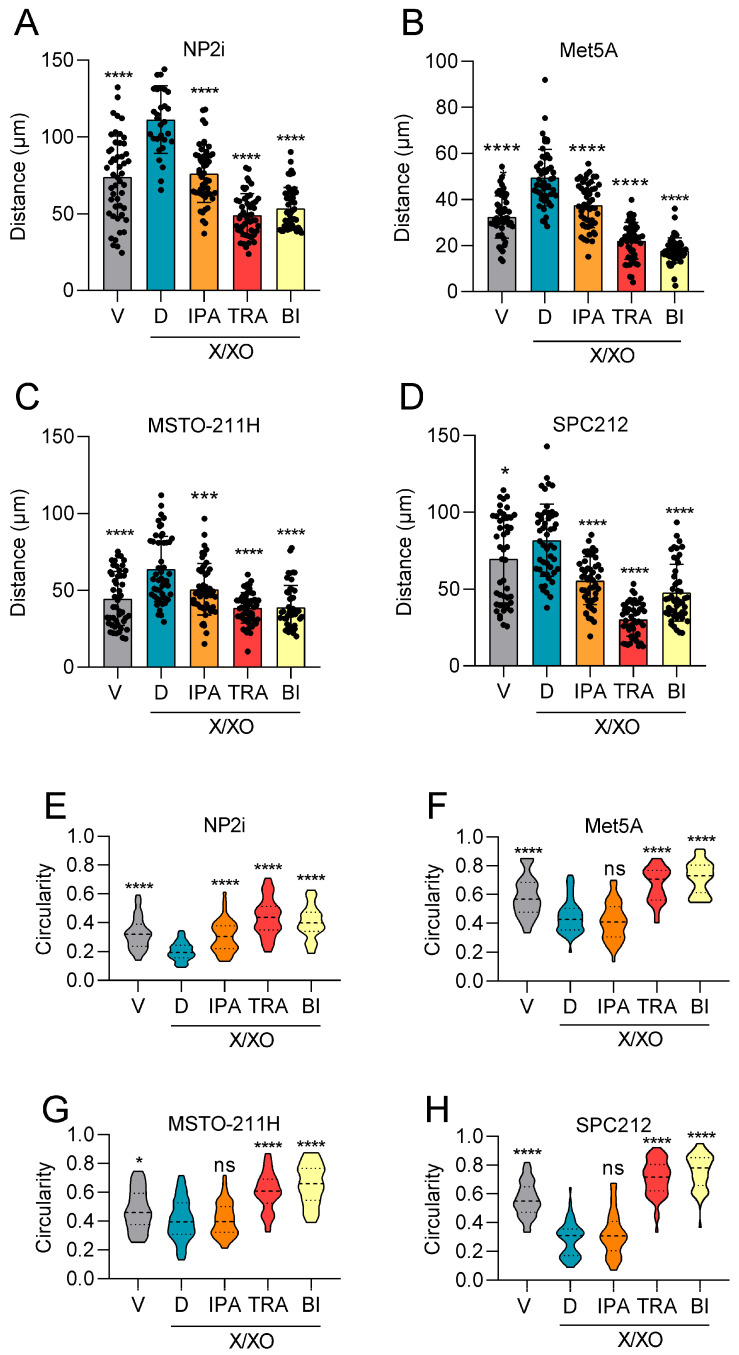
Pathway inhibitors reverse ROS-induced changes in cell migration and cell shape. Mesothelial (**A**,**B**) and pleural mesothelioma (**C**,**D**) cells were treated with xanthine/xanthine oxidase (X/XO, 2/20 µg/mL) or vehicle (V) plus where indicated with DMSO (D), ipatasertib (IPA, 10 µM), trametinib (TRA, 10 µM), or BI-D1870 (BI, 10 µM) for 24 h. Migrated distances of at least 50 cells were calculated from videomicroscopy data by manual tracking with Fiji/ImageJ and are shown as means (bars) and individual values of each cell (dots). Circularity of mesothelial cells (**E**,**F**) and pleural mesothelioma cells (**G**,**H**) treated as above for 24 h were calculated from microscopy images analysed with Fiji/imageJ, and data are shown as violin plots. Data from *n* = 3 independent experiments were analysed by one-way ANOVA followed by Dunnett’s test. Significance is indicated as ns *p* > 0.05, * *p* < 0.05, *** *p* < 0.001, and **** *p* < 0.0001, compared to DMSO control (D).

Having observed that ROS exposure results in increased cell migration and enhanced signalling through several pathways, we next explored whether inhibitors of the respective pathways could reverse these changes. We selected ipatasertib, a clinically investigated inhibitor of AKT [34], trametinib, a clinically approved inhibitor of MEK [35], and BI-D1870, an inhibitor of RSK, previously demonstrated by others and us to inhibit YB-1 phosphorylation [33,36]. Ipatasertib showed a relatively weak effect in NP2i and SPC212 cells and no significant effect in the other two cell models with respect to cell migration (Figure 4A–D). Trametinib showed a strong repression of cell migration in three of the four cell models (all except Met5A), whereas BI-D1870 strongly inhibited cell migration in all four cell models (Figure 4A–D). Direction autocorrelation curve and average cell speed calculation performed for NP2i and MSTO-211H also showed inhibitory effects by the pathway inhibitors (Supplementary Figure S6A–D). Similar effects to those on cell migration were observed when the circularity of cells was investigated, except that the ipatasertib effect was significant only in NP2i, and trametinib and BI-D1870 were both effective in all cell models (Figure 4E–H). Since RSK has multiple targets in addition to YB-1, we treated PM cells with SU056, which was previously described to directly bind and block YB-1 [37]. SU056 inhibited cell migration in both PM cell models, further supporting the link between YB-1 and cell migration (Supplementary Figure S7).

### 3.5. Trametinib and BI-D1870 Lead to a Strong Reduction in YB-1 Phosphorylation

**Figure 5:**
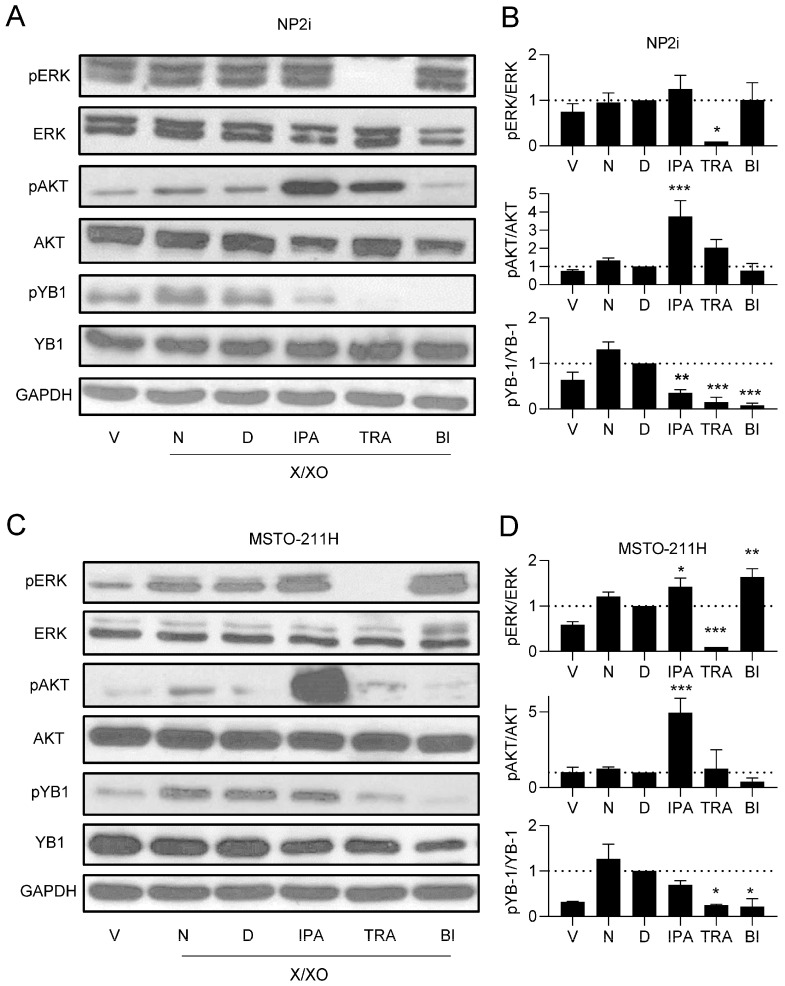
Effect of pathway inhibitors on ROS-induced phosphorylation of ERK, AKT, and YB-1. Representative examples (**A**,**C**) and quantification (**B**,**D**) of Western blots of phosphorylated (p) and total ERK, AKT, and YB-1 after treatment for 24 h with xanthine/xanthine oxidase (X/XO, 2/20 µg/mL) or vehicle (V) plus where indicated with DMSO (D), ipatasertib (IPA, 10 µM), trametinib (TRA, 10 µM), or BI-D1870 (BI, 10 µM) for 24 h. Lanes labelled as N represent cells treated with X/XO only. The housekeeping gene GAPDH was used as control for equal sample loading. Quantification data of *n* = 3 replicates are shown as phosphorylated-to-total protein ratios normalised to DMSO (D) samples set as 1. Data were analysed by one-way ANOVA followed by Dunnett’s test. Significance is indicated as * *p* < 0.05, ** *p* < 0.01, *** *p* < 0.001, inhibitor treated versus DMSO.

Next, we investigated the effect of the inhibitors on the respective signalling pathways. Notably, ipatasertib led to a strong increase in AKT phosphorylation in all cell models (Figure 5A–D, Supplementary Figure S8A–D). This effect likely resulted from reduced phosphatase accessibility induced by the binding of the ATP mimetic inhibitor and was previously reported for ipatasertib and other AKT inhibitors [38,39]. Ipatasertib also showed a slight decrease in pYB-1 levels in all cell models, in line with the notion that AKT contributes to the phosphorylation of YB-1 at the serine 102 position [40], which we have recently confirmed for PM cells [33]. Ipatasertib had either no or a slight increasing effect on pERK levels. In contrast, trametinib showed a complete inhibition of ERK phosphorylation and, in addition, a strong inhibition of pYB-1 levels in all cell models in agreement with its role as MEK inhibitor [35] and the notion that RSK activity is controlled by ERK [41] (Figure 5A–D, Supplementary Figure S8A–D). An increasing effect of trametinib on pAKT was seen only in NP2i but not in other models. The RSK inhibitor BI-D1870 had the strongest inhibiting effect on pYB-1 levels, with a weaker inhibiting effect on pAKT levels in all cell models but an increasing effect on pERK levels in three out of four models (all except NP2i) (Figure 5A–D, Supplementary Figure S5A–D).

### 3.6. ROS Treatment Increases the mRNA Expression of PD-L1 and PD-L2 but Not of TNFRSF9, PVR, or VISTA

**Figure 6:**
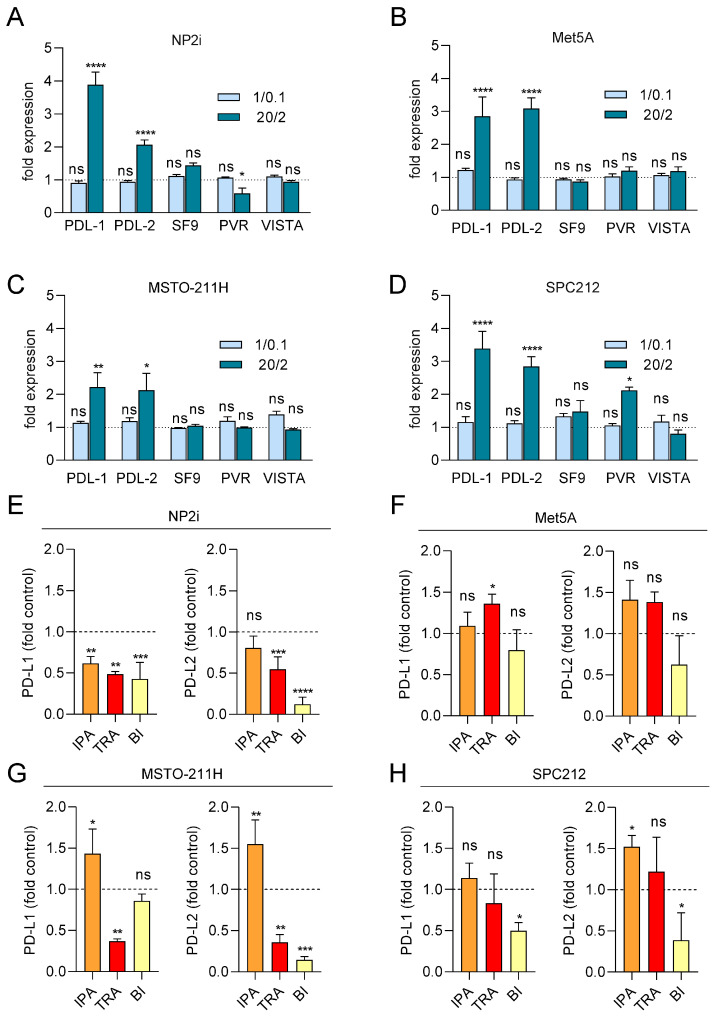
Expression of PD-L1 and PD-L2 mRNA is increased by ROS and partially decreased by pathway inhibitors. Mesothelial (**A**,**B**) and pleural mesothelioma (**C**,**D**) cells were treated with xanthine/xanthine oxidase (X/XO) at 1/0.1 and 20/2 µg/mL or vehicle, and gene expression levels of immune checkpoint proteins PD-L1, PD-L2, TNFRSF9 (SF9), PVR, and VISTA were determined using qRT-PCR and normalised to the housekeeping gene GAPDH. Bars represent fold expression values compared to vehicle-treated cells set as 1 and shown as horizontal line. Mesothelial (**E**,**F**) and pleural mesothelioma (**G**,**H**) cells were treated with 20/2 µg/mL X/XO plus either DMSO, ipatasertib (IPA, 10 µM), trametinib (TRA, 10 µM), or BI-D1870 (BI, 10 µM) for 24 h. Gene expression levels of immune checkpoint proteins PD-L1 and PD-L2 were determined using qRT-PCR and normalised to the housekeeping gene GAPDH. Bars represent fold expression values compared to X/XO plus DMSO-treated cells set as 1 and shown as horizontal line. Data from *n* = 3 experiments were analysed by one-way ANOVA followed by Dunnett’s test. Significance is indicated as ns *p* > 0.05, * *p* < 0.05, ** *p* < 0.01, *** *p* < 0.001, and **** *p* < 0.0001, X/XO versus vehicle in (**A**–**D**) and inhibitor treated versus DMSO in (**E**–**H**).

Since the expression of immune checkpoint proteins (ICPs) has been linked to cancer development, prognosis, and therapy response, we next investigated the impact of ROS on the transcript expression of several ICPs relevant to PM [42]. We analysed the mRNA expression levels of PD-L1, PD-L2, PVR, TNFRSF9, and VISTA after 24 h of ROS treatment using qRT-PCR. A striking effect was observed for PDL-1, which was increased by up to 4-fold in both mesothelial and PM cell models at the higher X/XO concentration (Figure 6A–D). Similarly, PD-L2 was significantly upregulated in both mesothelial and PM cells. The other ICPs showed less consistent effects, with PVR mRNA upregulated only in SPC212 but downregulated in NP2i after exposure to higher X/XO concentrations, and TNFRSF9 and VISTA mRNAs not significantly affected in any of the models (Figure 6A–D). 

### 3.7. PD-L1 and PD-L2 mRNA Expression Is Reduced by Trametinib and BI-D1870

Finally, we investigated the effect of signal pathway inhibition on PD-L1 and PD-L2 transcript expression under ROS treatment (Figure 6E–H). Ipatasertib showed a tendency to further increase both PD-L1 and PD-L2 mRNA expression in all models except NP2i. Trametinib and BI-D1870, in contrast, showed a tendency to reduce both PD-L1 and PD-L2 levels in the two PM cell models and one of the mesothelial cell models (NP2i).

## 4. Discussion

In this study, we show that ROS exposure resulted in changes in cell morphology and promoted cell migration in both mesothelial and PM cells. This was paralleled by the stimulation of the MAPK and AKT pathways and increased phosphorylation of YB-1. Moreover, ROS exposure upregulated the gene expression of PD-L1 and PD-L2 in mesothelial and PM cells, which could be inhibited by blocking RSK-mediated YB-1 phosphorylation. The ROS-mediated stimulation of cell migration has previously been shown in other cell types, e.g., prostate cancer cells [43]. In addition, ROS plays an important role in cell migration during wound healing [44]. However, data on the effect of ROS on the migration of mesothelial or PM cells have been lacking thus far. Since oxidative stress plays a key role in PM development [45], our data contribute to the understanding of PM biology.

The stimulation of migration by ROS in our study was associated with the activation of key cell signalling pathways. The activation of the MAPK pathway as well as the PI3K/AKT pathway by ROS is well documented in various cell types [46]. The role of YB-1 expression and phosphorylation in this context, however, has not yet been deciphered. Our research group has previously identified YB-1 as a relevant player in cell migration in PM cells via its ability to post-transcriptionally regulate Snail expression [27]. In addition, we have demonstrated that PM cell migration can be reduced by inhibiting YB-1 phosphorylation at the serine 102 position [33]. Furthermore, it has also been demonstrated that YB-1 expression is upregulated in PM cells compared to normal mesothelial cells in vitro and in tissue sections [12,33]. In the current study, we show that ROS exposure did not increase YB-1 expression in either mesothelial or PM cells; thus, ROS do not seem to contribute to the upregulation of YB-1 in PM. Nevertheless, a strong stimulation of YB-1 phosphorylation at serine 102 in response to ROS exposure was seen in the mesothelial and PM cells. RSK, which itself is phosphorylated by ERK and PI3K, has been shown to mediate YB-1 phosphorylation at serine 102 [47]. Therefore, we used an RSK inhibitor to block YB-1 phosphorylation. Our data clearly demonstrate that YB-1 is phosphorylated in response to ROS exposure, and that the inhibition of kinases upstream of YB-1 reduced both YB-1 phosphorylation and ROS-induced effects. However, RSK targets other than YB-1 [41] may also be required to mediate the effects observed with the RSK inhibitor. Our data on the YB-1 inhibitor SU056 [37] strengthen the link between YB-1 and cell migration in PM cell models. Nevertheless, additional work will be required to rule out the involvement of other RSK targets.

**Figure 7:**
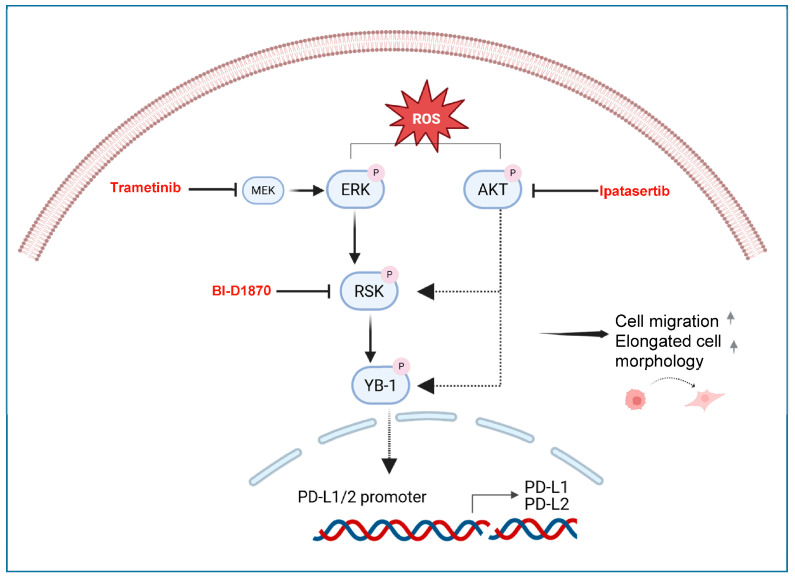
Proposed schematic model of ROS-induced effects in PM. ROS induce activation of MAPK and PI3KAKT signalling pathways. These pathways lead to phosphorylation of YB-1 and result in increased cell migration and elongated cell morphology. Phosphorylated YB-1 is hypothesised (indicated by dashed arrow) to translocate to the nucleus, where it may bind to promoters of PD-L1 and PD-L2, and increase their expression. YB-1 nuclear translocation and direct promoter binding were not experimentally tested in this study and are presented as a hypothetical model. Trametinib, ipatasertib, and BI-D1870 block or reduce YB-1 phosphorylation and reverse ROS-induced effects in mesothelial and PM cells. Created in BioRender. Michael Grusch (2025) https://app.biorender.com/illustrations/6901f752d95450e451d38357?slideId=412e7127-1090-4752-903d-4530d86cd771 (accessed on 13 January 2026) and the provided URL allows readers to access the high-resolution image directly.

Immune checkpoint proteins, especially PD-L1, have emerged as key therapeutic targets in PM and several other malignancies [19,48]. The expression of PD-L1 by tumour cells has been recognised as an important contributor to immune evasion during cancer development [18,19]. Our data suggest that the upregulation of PD-L1 and PD-L2 gene expression may already begin at an early stage of mesothelioma development when mesothelial cells are exposed to increased ROS levels. Differences in the regulation of expression have been described for PD-L1 and PD-L2 in macrophages and other cell types [49,50]. However, both ligands showed a remarkable similarity in their regulation in our models. Interferon gamma is considered one of the most prominent inducers of PD-L1 expression in many cell types [18,51]. Our group has previously described the upregulation of PD-L1 by FGF2 and EGF via the MAPK pathway in PM cells [52]. The impact of ROS on PD-L1 expression appears to be context-dependent, as both upregulation and downregulation have been reported [53]. In lung adenocarcinoma cell lines, for instance, it was demonstrated that iron-mediated oxidative stress increased PD-L1 expression in a c-Myc-dependent manner [22]. Another study in K-ras mutant pancreatic cancer described that ROS-mediated upregulation of PD-L1 expression depended on FGFR1 signaling [21]. In contrast, the elevated generation of mitochondrial ROS has resulted in the downregulation of PD-L1 via ubiquitination and subsequent degradation [54]. To the best of our knowledge, the impact of ROS exposure on PD-L1 expression in mesothelial cells or PM cells has not been described previously. Moreover, none of the existing studies on the regulation of PD-L1 by ROS has investigated a possible contribution of YB-1. Although ROS exposure does not increase YB-1 expression in our models, the observed stimulation of phosphorylation at serine 102 could lead to the enhanced translocation of YB-1 to the cell nucleus. This effect has been previously shown for PM cells, breast cancer cells, T cells, and T-cell acute lymphoblastic leukaemia cells [33,55,56]. In addition, several independent studies have established that nuclear YB-1 can bind to the PD-L1 promoter sequence and upregulate PD-L1 expression [15,16,17]. Combining these data from the literature with our current results, we propose a hypothetical model (Figure 7), in which ROS lead to the phosphorylation of YB-1 in PM cells via the MAPK/RSK pathway and, to a lesser degree, the PI3K pathway. Phosphorylated YB-1 then translocates to the nucleus and stimulates the transcription of PD-L1 and PD-L2. While the stimulation of PD-L1 transcription by YB-1 has been shown in the literature for lung cancer and hepatocellular carcinoma [15,16,17], the interaction of YB-1 with the PD-L2 promoter remains speculative at present. Noteworthily, YB-1 motifs are present upstream of the transcription start point and in the first intron of the PDCD1LG2 gene coding for PD-L2 (Supplementary Table S2).

Our study has several limitations. First, we do not have in vivo evidence and our data do not discriminate between the effects of superoxide anions and hydrogen peroxide, since both types of ROS are generated by the X/XO system. Another limitation is that ICPs were analysed only on the level of mRNA expression. Hence, further work will be required to confirm the link between ROS, (phospho)YB-1, and protein levels of ICPs in clinical specimens and animal models. If further confirmed, this mechanistic link could prove important for both therapeutic and preventive settings in PM. Reducing PD-L1 expression through blocking upstream signalling with kinase inhibitors could lead to enhanced antitumor immune responses in patients with PM. In this respect, it has been recently shown that CDKL1 can reduce the binding of YB-1 to the PD-L1 promoter in lung cancer models [17]. This resulted in downregulation of PD-L1 expression and enhanced the efficacy of radioimmunotherapy. Immunotherapy is increasingly used in patients with PM but shows only relatively modest response rates [20,57]. Therefore, combining PD-L1-targeting therapies with RSK inhibitors should be further investigated. The clinical utility of RSK inhibitors is currently being evaluated. A phase I/Ib/2 clinical trial is ongoing on the RSK inhibitor PMD-026 for breast cancer (NCT04115306).

In preventive settings, blocking immune-evasive signals may hold promise for slowing or preventing the development of PM. The long-term preventive administration of kinase inhibitors, such as trametinib or BI-D1870, may be problematic due to their side effects. However, healthier diets rich in antioxidants could be envisioned to reduce the ROS exposure of mesothelial cells and potentially reduce their PD-L1 expression and its immune-evasive capabilities.

In summary, we demonstrate that the ROS exposure of mesothelial and PM cells can contribute to enhanced cell migration and PD-L1 upregulation at the mRNA level. Our data provide a clear rationale for further exploring the link between ROS exposure, signalling pathway activation, and immune checkpoint protein expression during PM development.

## Data Availability

The original contributions presented in this study are included in the article/Supplementary Materials. Further inquiries can be directed to the corresponding author.

## Supplementary Materials

The following supporting information can be downloaded at: https://www.mdpi.com/article/10.3390/antiox15010121/s1. Table S1: Primers pairs used for qRT-PCR. Table S2: Potential YB-1 binding sites in the regulatory regions of the PD-L2 gene (*PDCD1LG2*). Figure S1: ROS treatment does not induce cytotoxicity in Met5A and SPC212 cells. Figure S2: ROS treatment-induced increase in migration shown in single-origin plots in Met5A, MSTO-211H, and SPC212 cells. Figure S3: ROS treatment-induces increased directional persistence and migration speed in NP2i and MSTO-211H cells. Figure S4: ROS treatment-induced effects on cell area and circularity in Met5A, MSTO-211H, and SPC212 cells. Figure S5: ROS treatment-induced effects on phosphorylation of ERK, AKT, and YB-1 in Met5A and SPC212 cells. Figure S6: Effects of pathway inhibitors on directional persistence and migration speed in X/XO-treated NP2i and MSTO-211H cells. Figure S7: Effect of the YB-1 inhibitor SU056 on pleural mesothelioma (PM) cell migration. Figure S8: Effects of pathway inhibitors on phosphorylation of ERK, AKT, and YB-1 in Met5A and SPC212 cells.

## Abbreviation

The following abbreviations are used in this manuscript:

AKT	Protein kinase B
BAP1	BRCA1-associated protein 1
BF	Bright field
CDKN2A	Cyclin-dependent kinase inhibitor 2A
DCFDA	2′,7′-Dichlorodihydrofluorescein diacetate
EGF	Epidermal growth factor
EGFR	Epidermal growth factor receptor
ERK	Extracellular signal-regulated kinase
FGF2	Fibroblast growth factor 2
FGFR1	Fibroblast growth factor receptor 1
GFP	Green fluorescent protein
MAPK	Mitogen-activated protein kinase
MEK	Mitogen-activated protein kinase (MEK1/2)
MTAP	Methylthioadenosine phosphorylase
NP2i	Immortalised hTERT transduced normal pleural mesothelial cell
PD-L1	Programmed death ligand 1
PD-L2	Programmed death ligand 2
PM	Pleural mesothelioma
ROS	Reactive oxygen species
RSK	p90 ribosomal S6 kinase
TP53	Tumour protein 53
X	Xanthine
XO	Xanthine oxidase
YB-1	Y-box binding protein 1
