# Supplementary material for: Reactive Oxygen Species Drive Cell Migration and PD-L1 Expression via YB-1 Phosphorylation in Pleural Mesothelioma
Source: Antioxidants (Basel). 2026 Jan 17;15(1):121. doi: 10.3390/antiox15010121 (PMC12838097; doi:10.3390/antiox15010121)
Supplement: Supplementary file 1 [file antioxidants-15-00121-s001.zip › antioxidants-4004026-supplementary.pdf]

Supplementary Table S1: Primer pairs used for qRT-PCR

Gene	Primer Type	Sequence (5'–3')	Reference
**PD-L1**	Forward	TGGTCATCCCAGAACTAC	This study
**PD-L1**	Reverse	GATGGCTCCCAGAATTAC	This study
**PD-L2**	Forward	CTGTGGCAAGTCCTCATATC	This study
**PD-L2**	Reverse	TGCAATTCCAGGCTCAAC	This study
**PVR**	Forward	CACTGTCACCAGCCTCTGGATA	Origene (HP209435)
**PVR**	Reverse	TCATAGCCAGAGATGGATACCTC	Origene (HP209435)
**VISTA**	Forward	AGATGCACCATCCAACTGTGTGG	OriGene (HP214399)
**VISTA**	Reverse	AGGCAGAGGATTCCTACGATGC	OriGene (HP214399)
**TNFRSF9**	Forward	TCGACCCTGGACAAACTG	This study
**TNFRSF9**	Reverse	CTCTCCTTCGTCCCATTCAC	This study
**GAPDH**	Forward	CACCGTCAAGGCTGAGAAC	This study
**GAPDH**	Reverse	TCTCGCTCCTGGAAGATGG	This study

Supplementary Table S2: Potential YB-1 binding sites in regulatory regions of the PD-L2 gene (PDCD1LG2)*.

Motif ID	Alt ID	Sequence Name	Strand	Start	End	p-value	q-value	Matched Sequence
**UN0139.1**	YBX1	FP013058	-	-1406	-1398	3.22e-05	0.546	CGTCCCATC
**UN0139.1**	YBX1	FP013058	+	6198	6206	3.95e-05	0.546	AGTACCATC
**UN0139.1**	YBX1	FP013058	-	7344	7352	5.91e-05	0.546	TGCTCCATC

*The Y-box binding protein 1 (YBX1) DNA-binding motif (UN0139.1) was retrieved from the JASPAR (Joint Annotated Sequence Platform for Analysis of Regulatory elements, https://jaspar.elixir.no/) database and used as input for FIMO (Find Individual Motif Occurrences, https://meme-suite.org/meme/doc/fimo.html). The genomic sequence from -2000 to 12000 of the transcription start point of the PDCD1LG2 gene including upstream regulatory regions and the complete intron 1 was downloaded from EPD (The Eukaryotic Promoter Database, https://epd.expasy.org/epd/) and used as the target sequence for scanning in FIMO. Statistically significant motif occurrences based on FIMO-calculated p-values are shown relative to the transcription start point.

.

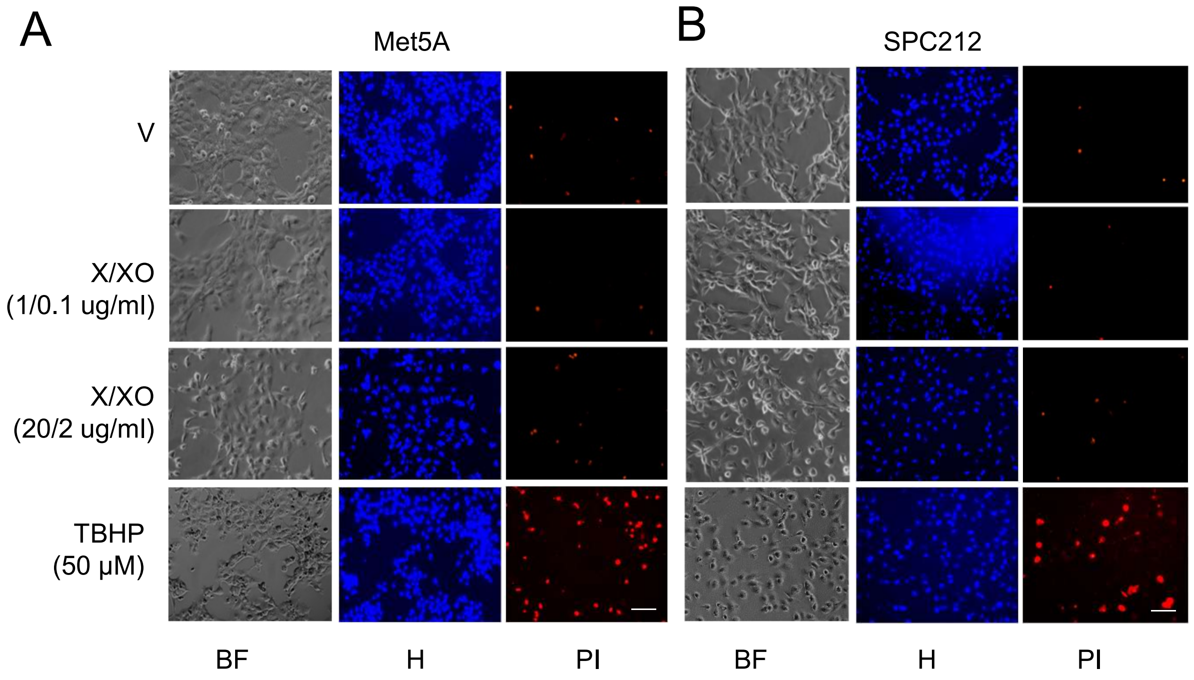

Supplementary Figure S1: ROS treatment does not induce cytotoxicity. Double staining with Hoechst 33342 and propidium iodide (PI) of mesothelial cells (A) and pleural mesothelioma cells (B) treated with vehicle (V) or 1/0.1 µg/ml and 20/2 µg/ml X/XO for or 50 µM TBHP (as positive control) 24 hours. Hoechst 33342 (blue) shows the nuclear morphology, propidium iodide (PI, red) labels dead cells that have lost membrane integrity. Green fluorescence protein (GFP) and brightfield (BF) images are shown for NP2i and MSTO-211H cells, respectively. All microscopic images were taken at same magnification. Scale bar = 50 µm.

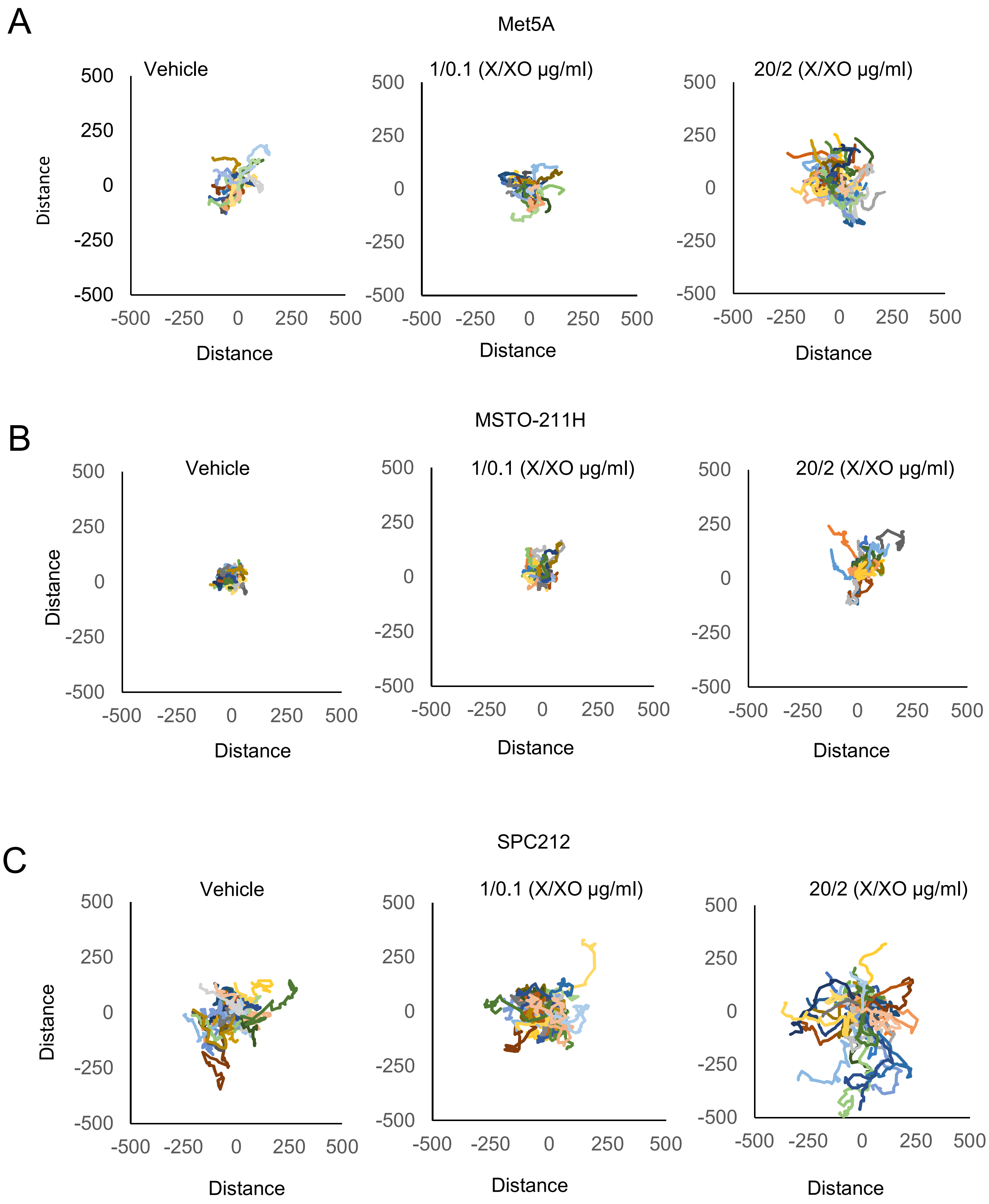

Supplementary Figure S2: ROS treatment-induced increase of migration shown in single-origin plots. Mesothelial cells (A) and pleural mesothelioma cells (B, C) were treated with xanthine/xanthine oxidase (X/XO) at the indicated concentrations or vehicle for 24 hours and single origin plots of representative cells were generated from videomicroscopy data with Image J and the DiPer origin plot module. Data are from n = 3 experiments.

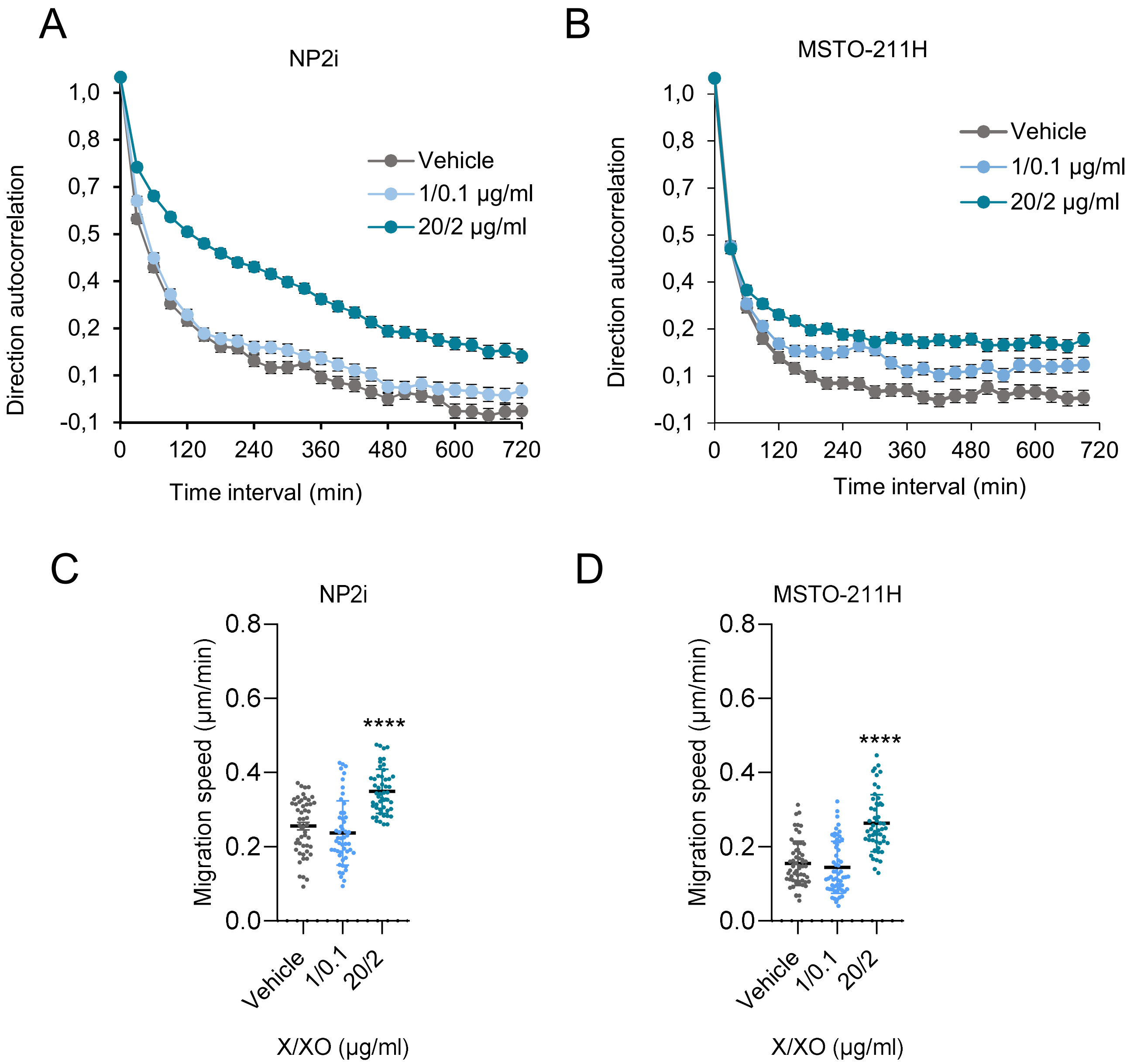

Supplementary Figure S3: ROS treatment-induces increased directional persistence and migration speed in NP2i and MSTO-211H. Direction autocorrelation over time of migrating cells treated with vehicle, X/XO (1/0.1 µg/ml), or X/XO (20/2 µg/ml) (A, B). Direction autocorrelation curves were calculated from cell trajectories using the DiPer autocorrelation module; higher values and slow decay indicate enhanced directional persistence. Single-cell average migration speeds were calculated from the same trajectories using the DiPer speed module (C, D). Each dot represents an individual cell, and horizontal lines indicate the mean ± SD of all 50 cells from n = 3 experiments analyzed per condition. Data were analysed by one-way ANOVA followed by Dunnett's test. Significance is indicated as **** $p < 0.0001$, X/XO versus vehicle.

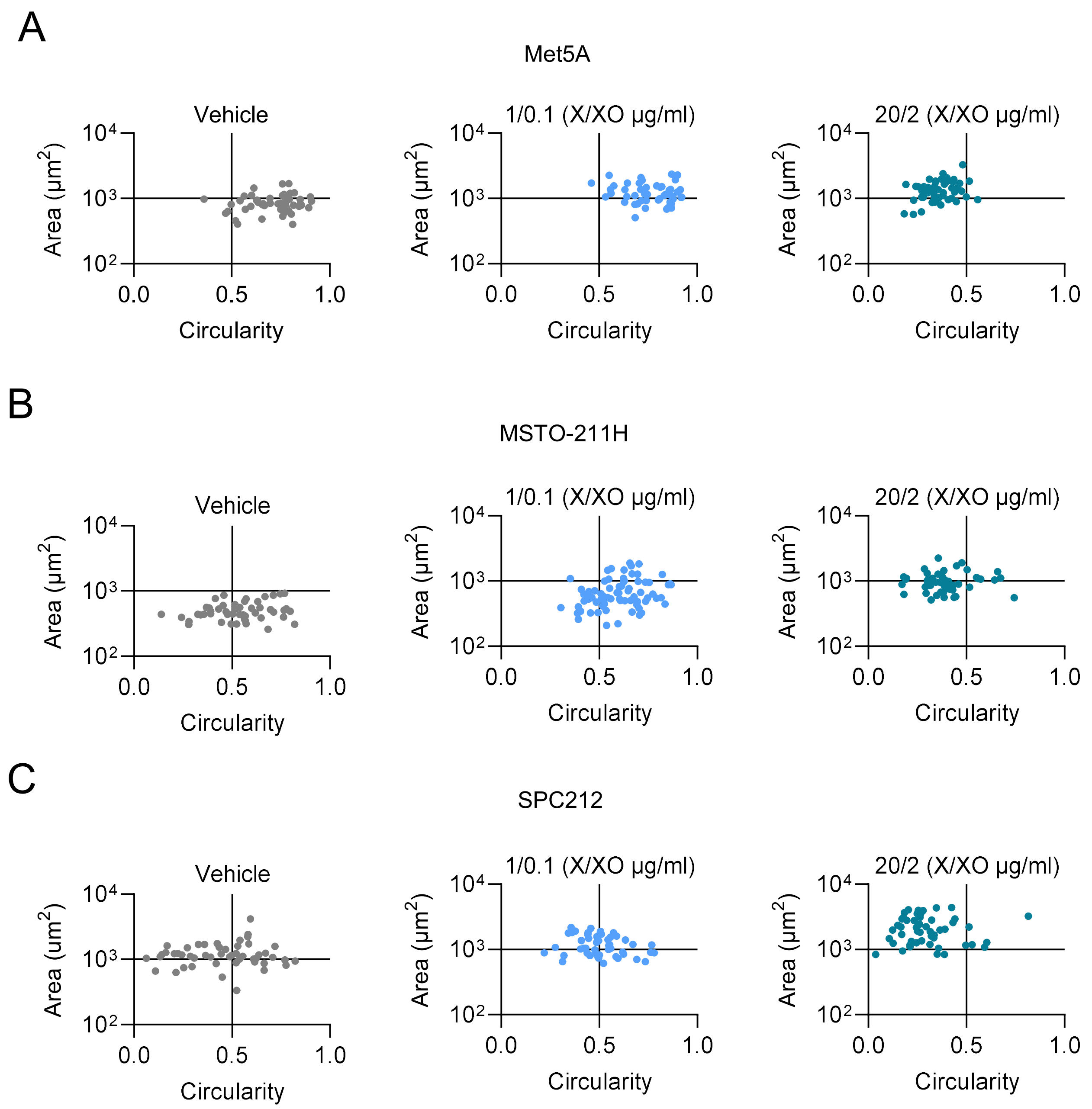

Supplementary Figure S4: ROS treatment-induced effects on cell area and circularity. Mesothelial cells (A) and pleural mesothelioma cells (B, C) were treated with xanthine/xanthine oxidase (X/XO) at the indicated concentrations or vehicle. Cell shape descriptors (area, circularity) of at least 50 individual cells were determined with image J from microscopy images obtained at the end of the 24 hour treatment period. Data are from n = 3 experiments.

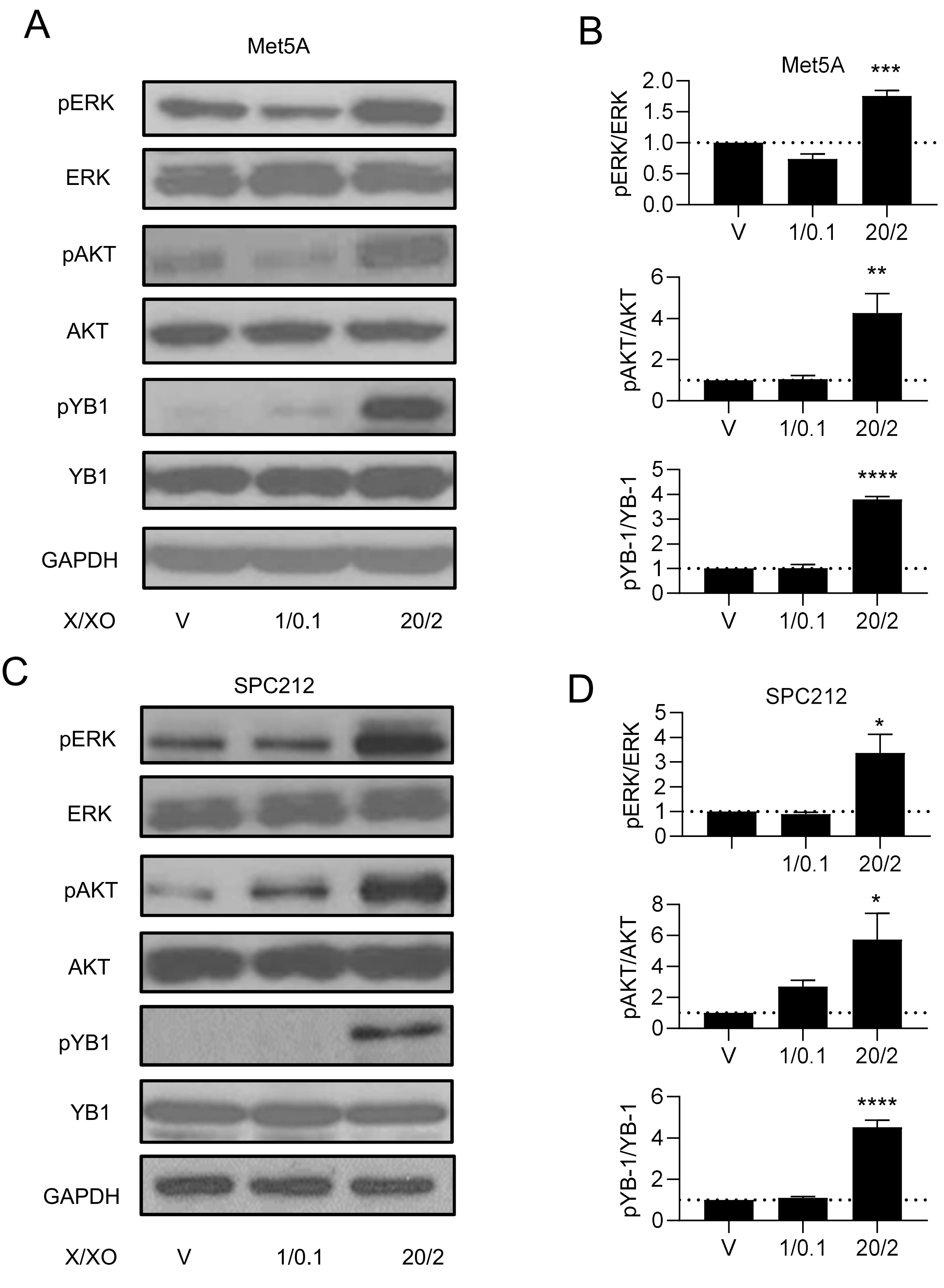

Supplementary Figure S5: ROS treatment-induced effects on phosphorylation of ERK, AKT and YB-1. Mesothelial cells (A, B) and pleural mesothelioma cells (C, D) were treated with xanthine/xanthine oxidase (X/XO, µg/ml) at the indicated concentrations or vehicle (V) and subjected to Western blot analysis. GAPDH was used as loading control. Representative examples (A,C) and quantification (B,D) of phosphorylated (p) to total protein ratios normalized to vehicle are shown. Quantification data from n = 3 experiments are shown as phosphorylated-to-total protein ratios normalized to vehicle-treated samples set as 1. Data were analysed by one-way ANOVA followed by Dunnett's test. Significance is indicated as * $p < 0.05$, ** $p < 0.01$, *** $p < 0.001$, and **** $p < 0.0001$, X/XO versus vehicle.

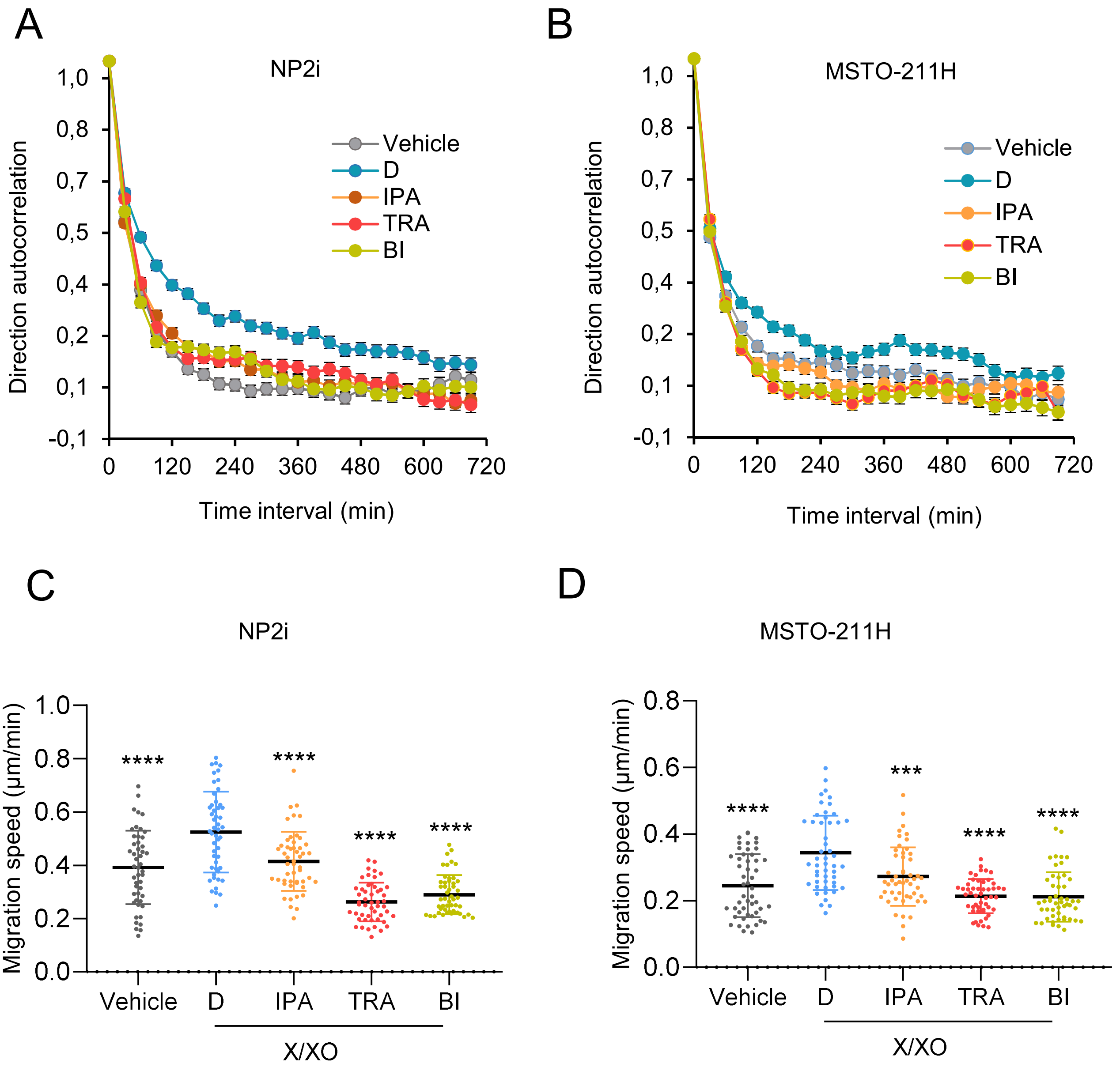

Supplementary Figure S6: Effects of pathway inhibitors on directional persistence and migration speed in X/XO-treated NP2i and MSTO-211H cells. Direction autocorrelation over time of migrating cells treated with vehicle or X/XO (20/2 µg/ml) plus DMSO (D), ipatasertib (IPA, 10 µM), trametinib (TRA, 10 µM) or BI-D1870 (BI, 10 µM) (A, B). Direction autocorrelation curves were calculated from cell trajectories using the DiPer autocorrelation module. Single-cell average migration speeds were calculated from the same trajectories using the DiPer speed module (C, D). Each dot represents an individual cell, and horizontal lines indicate the mean ± SD of all 50 cells from n = 3 experiments analysed per condition. Data were analysed by one-way ANOVA followed by Dunnett's test. Significance is indicated as *** $p < 0.001$, **** $p < 0.0001$ versus D.

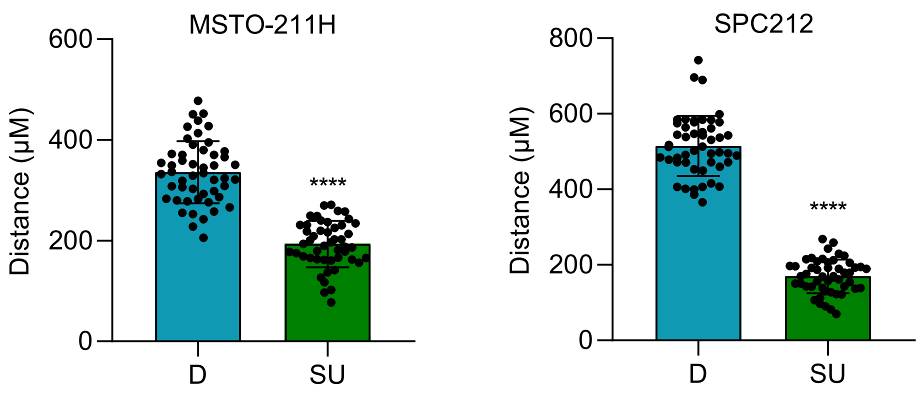

Supplementary Figure S7: Effect of the YB-1 inhibitor SU056 on PM cell migration. Pleural mesothelioma cells (MSTO-211H and SPC212) were treated with DMSO (D) or SU056 (SU, 3 µM) for 72 h. Migrated distances of at least 50 cells from n = 3 experiments were calculated from videomicroscopy data by manual tracking with Fiji/ImageJ and are shown as means (bars) and individual values of each cell (dots). Data were analysed by unpaired t-test. Significance is indicated as **** $p < 0.0001$ SU056-treated versus DMSO-treated cells.

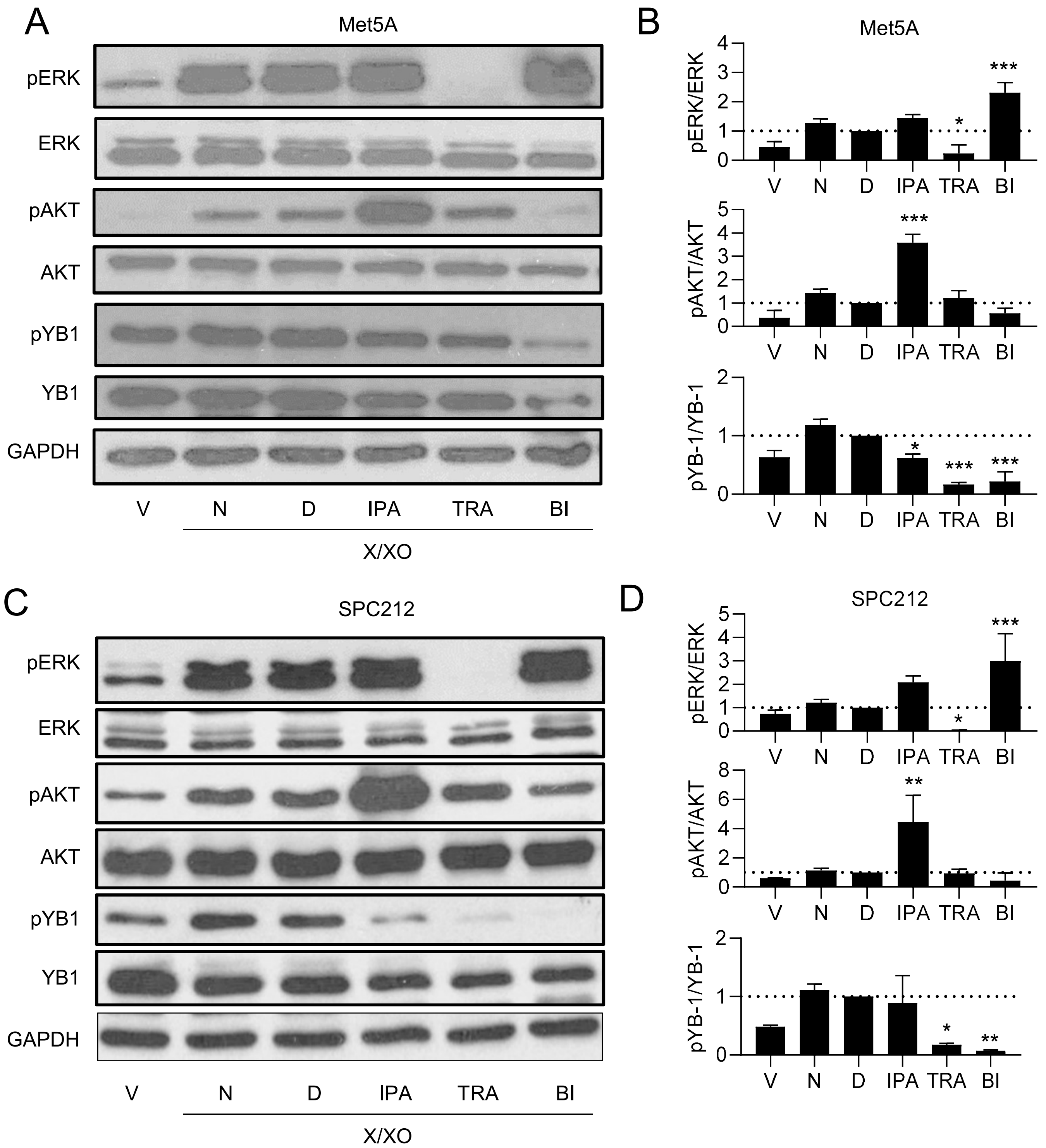

Supplementary Figure S8: Effect of pathway inhibitors on phosphorylation of ERK, AKT and YB-1. Mesothelial cells (A, B) and pleural mesothelioma cells (C, D) were treated with xanthine/xanthine oxidase (X/XO, 20/2 µg/ml) or vehicle (V) plus DMSO (D), ipatasertib (IPA, 10 µM), trametinib (TRA, 10 µM) or BI-D1870 (BI, 10 µM) for 24 hours and subjected to Western blot analysis. GAPDH was used as loading control. N represents samples treated with X/XO only. Representative examples (A,C) and quantification (B,D) of phosphorylated (p) to total protein ratios normalized to DMSO controls (D) are shown. Quantification data of n = 3 replicates are shown as phosphorylated-to-total protein ratios normalized to vehicle-treated samples set as 1. Data were analysed by one-way ANOVA followed by Dunnett's test. Significance is indicated as * $p < 0.05$, ** $p < 0.01$, *** $p < 0.001$, inhibitor treated versus DMSO.
